# Proteins from the DNA Damage Response: Regulation, Dysfunction, and Anticancer Strategies

**DOI:** 10.3390/cancers13153819

**Published:** 2021-07-29

**Authors:** Caroline Molinaro, Alain Martoriati, Katia Cailliau

**Affiliations:** Univ. Lille, CNRS, UMR 8576-UGSF-Unité de Glycobiologie Structurale et Fonctionnelle, F-59000 Lille, France; caroline.molinaro@univ-lille.fr (C.M.); alain.martoriati@univ-lille.fr (A.M.)

**Keywords:** DNA damage response, DNA damage therapy, DNA repair, DDR inhibitors, cell cycle, cancers

## Abstract

**Simple Summary:**

Cells respond to genotoxic stress through complex protein pathways called DNA damage response (DDR). These mechanisms ensure the preservation of genomic integrity and activate DNA repair, cell cycle regulation, and, ultimately, programmed cell death. When altered, the DDR protein network leads to several diseases, particularly cancers. In recent years, the vulnerabilities of the DDR network have been successfully exploited to improve cancer treatments using DNA damage strategies and therapies combination.

**Abstract:**

Cells respond to genotoxic stress through a series of complex protein pathways called DNA damage response (DDR). These monitoring mechanisms ensure the maintenance and the transfer of a correct genome to daughter cells through a selection of DNA repair, cell cycle regulation, and programmed cell death processes. Canonical or non-canonical DDRs are highly organized and controlled to play crucial roles in genome stability and diversity. When altered or mutated, the proteins in these complex networks lead to many diseases that share common features, and to tumor formation. In recent years, technological advances have made it possible to benefit from the principles and mechanisms of DDR to target and eliminate cancer cells. These new types of treatments are adapted to the different types of tumor sensitivity and could benefit from a combination of therapies to ensure maximal efficiency.

## 1. Introduction

The genome is constantly harmed by spontaneous damage caused by endogenous factors produced by normal cellular physiological conditions such as bases alteration, aberrant DNA enzyme function or oxidation, and by a large variety of exogenous genotoxic factors [1]. Cells have evolved a complex network of hundreds of proteins, named the DNA damage response (DDR), to ensure genome integrity and the expression of dedicated proteins to each cell type. The quality control mechanisms of DDR senses DNA damage, coordinates DNA repair with cell cycle arrest, and ensures cell death when repairs are not possible [2]. The detection of DNA damage involves the recruitment of various repair protein complexes depending on the type of break. Classically, phosphatidylinositol-3-kinase-related kinases ATM (ataxia-telangiectasia mutated), ATR (ATM- and Rad3-related), and DNA-PKcs (DNA-dependent protein kinase, catalytic subunit) are activated [3]. These kinases phosphorylate numerous targets at the DNA damage sites including CHK1/2 (checkpoint kinases 1/2) and histone H2AX. Substrates of CHK kinases are effectors for DNA repair, transcription, and cell-cycle control, such as BRCA1, NBS1, P53, CDC25, and CDKs (cyclin-dependent kinase). The cellular outcome depends on the types but also on the severity of DNA damage, the cell cycle state, chromatin modifications, post-translational events, and non-coding RNA. Cells with excessive or unrepairable DNA undergo an apoptotic P53-dependent death or another type of programmed cell death that does not rely on caspase activation.

Many natural cellular events rely on DNA breaks/repairs and use part of the DDR network to fulfill specific physiological functions. Despite physical and chemical agents, several biological agents induce severe DNA damage. When DDR processes are damaged or bypassed, DNA damage elicits mutations and heritable changes resulting in pathologies (e.g., immunodeficiency, inflammation, neurodegeneration, aging, cardiovascular diseases, and cancer). When and how the DDR complex signaling network of proteins is controlled to ensure the right cell outcome; and how the vulnerabilities are exploited in precision medicine to target and treat cancers (from single inhibitor to combined treatments) are hereby highlighted and updated in the present review.

## 2. DDR Proteins Activation Is Function of the Type of DNA Damage

**Figure 1:**
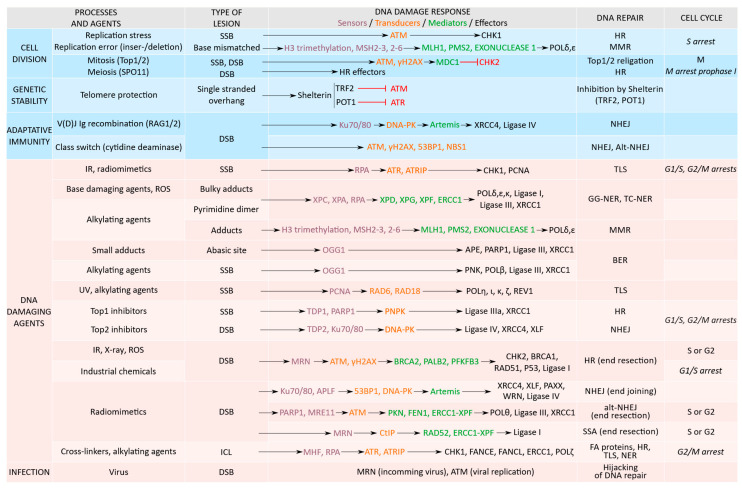
Classification of proteins from the DNA repair processes according to the DNA damaging agents, the type of DNA damage in relation with recruited effectors, repair mechanisms, and cell cycle phases occurrence or induced arrests. DNA damaged proteins are classified as sensors (violet), transducers (orange), mediators (green), and effectors (black). Natural processes, showed in the upper blue part, display a non-canonical DDR. They inhibit effectors, restrain the DNA repair in mitosis and protects telomere ends (inhibited proteins in red). Canonical DDR pathways are shown in the pink lower part.

Cells encounter tens of thousands of DNA lesions every day arising from endogenous cellular functions and exogenous environmental factors. Intracellular and external DNA damaging events create more than a single type of lesion. DNA damage inflicted to the DNA’s double helix includes base damage that does not involve breakage of the phosphodiester backbone or single-strand DNA breaks (SSBs) and double-strand breaks (DSBs). Single base alterations are generated by depurination, deamination, alkylation (usually by guanine methylation), oxidation (production of 8-oxo-7, 8-dihydroguanine), hydrolysis or chemical bonds cleavage in DNA, base analog incorporation, and stable covalent DNA adducts formation. Two-base alterations are formed by a thymine-thymine dimer or by cross-linking under the effect of a bifunctional alkylating agent. DNA strand breaks arise from oxidative or DNA replication stress, transcriptional stalling, failure to repair processes, and abnormal high effectors activation [4,5]. SSBs are converted into DSB lesions during DNA replication [6] (Figure 1).

### 2.1. Replication Stress and DNA Damage

DNA replication disturbances or the slowing/stalling of the replication fork progression during DNA synthesis generate replication stress. Replication stress is a source of massive DNA damage. It arises as a consequence of normal cellular reactions involving DNA, or upon exposure to external agents. Exogenous factors affecting DNA are numerous, ranging from physical agents such as ionizing radiation to genotoxic chemicals (organic compounds such as aromatic substances, or inorganic compounds such as heavy metals [7]) and biological agents including pathogens (bacteria, DNA, and RNA viruses), fungi, and plants [8,9,10]. Those agents also increase the ROS formation that enhances DNA damage [11].

The endogenous factors responsible for replication stress are numerous. DNA sequences can form special structures such as secondary hairpin loops, G-quadruplexes, display damage, or replication/transcription conflict, R-loops, topological stress constraints, or dysregulation of replicative proteins such as RPA, a natural barrier for replication forks [12]. Imbalance in the cellular pool of dNTPs also affect DNA polymerases inactivation and require two different RAD51-mediated pathways for restart and repair [13].

Damaged DNA bases, as a consequence of natural reactions such as depurination, base oxidation, and bulky adducts, are not recognized as templates by DNA polymerases, creating a source of replication stress [14]. On the contrary, interstrand crosslinks do not generate extensive areas of ssDNA (single stranded DNA) and are repaired by the FA (Fanconi Anemia) pathway [15].

Replication forks operate on genomic regions with special chromatin structures such as telomeres and centromeres that represent a challenge for the replisome. R-loops, formed by a DNA/RNA duplex and a ssDNA, arise during transcription and replication-transcription collision. They promote transcription-associated recombination and genome instability in mitotic and meiotic cell cycles [16] and the exposed ssDNA is processed into a DSB by transcription-coupled NER (nucleotide excision repair) [17].

Replication/transcription collisions can lead to transcription-associated recombination and chromosomal rearrangements. To minimize these encounters, both processes are temporally and spatially separated in different cell cycle phases S and G1 [18]. The THO/TREX (TRanscription-Export) and THSC/TREX-2 complexes that mediate the processing of mRNAs and nuclear export are required to avoid replication-transcription conflicts and may prevent R-loop formation [19].

### 2.2. Physiological DNA Breaks Involved in Normal Cellular Processes

Several natural events, necessary for cellular integrity, require programmed DNA lesions and a partial DDR recruitment [20] (Figure 1).

The replication stress checkpoint enables the DDR to act in coordination with the replisome to protect the stalled forks from degradation and to restart broken forks. Replication forks stalling triggers the activation of the ATR-CHK1 pathway for stabilization and is followed by the release of CHK1 from the chromatin, allowing for the phosphorylation and degradation of CDC25A and cell cycle S phase arrest [21,22,23].

During the course of the cell cycle, in the replicative and the mitosis phases, DNA is naturally cut and religated by topoisomerases (Top) to control DNA topology. To allow DNA or RNA polymerase action, topoisomerases (Top1 and 2) bind to DNA, cut the phosphate backbone of, respectively, one or two strands (SSB or DSB), and untangle DNA before the DNA backbone is resealed. Mitotic DDR functions to coordinate microtubule/kinetochore attachment and spindle assembly [24]. Top2 is necessary for chromosomal segregation before anaphase, and for chromosomal structure maintenance [25]. In mitosis, damage of condensed chromosomes activate initial upstream effectors such as H2AX-MDC1 (mediator of DNA damage checkpoint 1) platform, and the downstream signaling cascade is restrained (inhibition of CHK2), waiting for further progression into the cell cycle [26,27].

DNA lesions also occur through enzymatic reactions and chemical modifications from intracellular metabolism, the release of free radicals, reactive oxygen species (ROS), reactive nitrogen species (RNS), and reactive carbonyl species (RCS) that trigger a chain of reactions and result in a production of exocyclic adducts to DNA bases [28].

The production of male and female gametes for sexual reproduction involves a natural DSB formed during the meiotic recombination process by the SPO11 topoisomerase [29,30]. Meiotic DSBs occur in prophase from the leptotene to the pachytene stage of the first meiotic division. They are essential to allow chromosomal DNA exchange between non-homologous sister chromatids when they are paired to create genetic diversity [31]. A failure in this process directs germ cells to aneuploidy and leads to congenital disorder or pregnancy loss. During spermatogenesis, when histones are substituted by protamines, Top2 generates temporal DNA breaks to relax DNA that needs to be repaired properly before the end of spermiogenesis to avoid mature spermatozoa with fragmented DNA [32,33]. During meiotic sex chromosome inactivation (MSCI) in males, the sequestration of DDR factors (ATR and H2AX histone) to the XY chromatin away from the autosome is critical for meiosis progression [34].

The variable exon regions encoding the antigen recognition sites of B and T lymphocyte’s receptors, respectively involved in humoral and cellular-mediated immunity, are generated by a V (variable), D (diversity), or J (joining) genes recombination during early development. In response to an antigen, class switch recombination modifies the immunoglobulin genes in B lymphocytes [35]. V(D)J recombination starts by the formation of a DSB in DNA by RAG1 and RAG2 (recombination activating gene 1/2) proteins and are repaired through the recruitment of the non-homologous end-joining (NHEJ) pathway composed by Ku70/80, DNA-PK, XRCC4 (X-ray repair cross-complementing protein 4)/DNA-ligase IV, and Artemis [36,37,38]. The class switch recombination (CSR) breaks are initiated in B-cells by a specific activation-induced cytidine deaminase (AID) that mediates DNA deamination. Unlike the V(D)J recombination, the CSR depends on non-canonical BER and MMR to generate DSBs that are finally joined by NHEJ for blunt ends and alt-NHEJ for staggered breaks. DDR factors such as H2AX, ATM, 53BP1, and NBS1 are also involved in CSR [39]. CSR and V(D)J recombination represent a paradigm to study regulations among the various DNA repair pathways. A SAGA deubiquitination module was recently shown to promote DNA repair and class switch recombination through DNA-PK and ATM-induced γH2AX formation [40]. These proteins were found to function upstream of various double-stranded DNA repair pathways. Interestingly, IgG were found to be produced in non-immune cells (spermatozoids) through CSR with RAG1/2 and AID, suggesting a role in fertilization, but also that gametes are far more complex in their DDR regulation [41].

Telomeres located at chromosomal ends protect chromosomes from end-to-end fusions and abnormal mitosis segregation. Telomeric DNA presents a single-stranded overhang protected by a specialized capping complex, called shelterin, composed by TRF1 and TRF2 (telomere repeat-binding factor 1 and 2), TIN2 (TRF1-interaction factor 2), RAP1 (repressor activator protein 1), TPP1, and POT1 (protection of telomere 1), that blocks the recognition by the DDR repair machinery [42]. After each replication, telomeres are partly deprotected, recruit a non-canonical DDR in the G2 phase of the cell cycle, and reconstruct the end protection complex. Only the MRN complex (MRE11/RAD50/NBS1) and the ATM kinase are activated, while the downstream diffusible DDR signaling proteins, CHK2 and P53, are not, avoiding downstream cell-cycle inhibition [43].

## 3. Structural Organization of the DDR

The type of ends and the structures of the DNA breaks dictate the selection and the recruitment of repair factors. The choice of the cell response and its outcome depends on factors from the DNA repair pathway, the cell cycle checkpoints, the expression level of effectors from the P53 family, the chromatin state, the genomic location of the break, the post-translational modifications undergone by cytoplasmic or nuclear proteins, and RNA metabolism [44,45].

### 3.1. Selection of the Repair Mechanisms

After the detection of DNA damage, repair mechanisms are activated depending on the break types (Figure 1). The critical steps of the DNA repair process are the recognition and the signaling of the DNA lesions. DNA breaks can be repaired by direct reversal of the chemical reaction of the DNA damage using O^6^-methylguanine methyltransferase (MGMT), or by removal and replacement of the damaged bases [46]. Repair processes involve an important number of effectors, multiple interactions, and post-translational modifications [47,48].

For SSBs, the following three main excision repair pathways exist: NER (nucleotide excision repair), BER (base excision repair), and MMR (mismatch repair). NER includes XPC/A key components for the recognition of the DNA lesion, ERCC1, ERCC4 (XPF), and XPG for excision (excision repair cross-complementation group 1/4/6), recruitment of PCNA (proliferating cell nuclear antigen) by RFC (replication factor C) to allow the copy of the undamaged strand by DNA polymerases (δ, ε, and/or κ), and finally ligated by the ligase-I or ligase-III-XRCC1 complex [49]. The repairs of the lesions throughout the genome are processed by GG-NER (global genome NER) and those blocking transcription by TC-NER (transcription coupled NER). BER is activated for bulky adducts. BER starts with OGG1 (8-Oxoguanine glycosylase), forming abasic (AP) sites cleaved by AP endonucleases, resulting in single-strand breaks further processed by single nucleotide replacement or synthesis and by the recruitment of PARP1/2, XRCC1, ends with polymerase β and ligase-I and III. The MMR, or strand-specific mechanism, recognizes and repairs erroneous insertion, deletion, or miss-paired bases, includes MSH (MutS Homolog) and MLH/PMS proteins. A translesion synthesis process (TLS) allows the DNA replication machinery to bypass DNA lesions such as AP sites or thymine dimers. In TLS, PCNA is ubiquitinated by RAD6 and RAD18 and allows the binding of polymerases (η, ι, κ, ζ, REV1) [50].

DSB repairs are processed by a non-homologous end joining (NHEJ), an alt-NHEJ also named micro-homology mediated end joining (MMEJ), single-strand annealing (SSA), or by a homologous directed repair (HDR) including the homologous recombination (HR). NHEJ mobilized in the G1 phase of the cell cycle requires the recognition of DNA damage by Ku70/80, the recruitment of APLF (Aprataxin and PNK-like factor), 53BP1 (P53-binding protein 1), DNA-PK, end processing by Artemis, the recruitment of PAXX (paralogue of XRCC4 and XLF) and polymerase δ, µ, and ligation by ligase-IV with partner XRCC4 and XLF (XRCC4-like factor). Alt-NHEJ/MMEJ is initiated by PARP1 and MRE11 nuclease to resect DNA-ends that generate single-stranded overhangs annealed at microhomologies and recruits polymerase θ. SSA requires RAD52 for annealing in addition to the ERCC1/4 endonucleases complex and ligase I. HR is activated in the S and G2 phases of the cell cycle and requires the ATM-MRN complex, CtIP (CtBP-interacting protein), RPA (replication protein A), BRCA1 (breast cancer 1), and PALB2 (partner and localizer of BRCA2) recruitment of BRCA2 that acts with RAD52 to mobilize RAD51 and PFKFB3 (6-phosphofructo-2-kinase/fructose-2,6-biphosphatase 3). In case of DSBs, CHK2 phosphorylates BRCA1, serving not only as a scaffold protein but also as a clock for HR repair [51]. The selection between the different DSB repair processes is, in part, driven by the length of the homologies [52,53].

Interstrand crosslinks (ICLs) are DNA strands linked with covalent bonds preventing the separation of DNA strands that are repaired by the FA (Fanconi anemia) pathway using FANCs proteins and effectors from the NER, TLS, HR, factors responsible for Fanconi anemia, and structure-specific endonucleases [54].

During gametogenesis, the DDR has a specific organization. In spermatogenesis, HR acts during the S phase to resolve the DNA damage and NHEJ in G1. Haploid spermatids use an Alt-NHEJ pathway due to the absence of the main components of the canonical NHEJ. Spermatozoa possess a truncated BER containing only the first enzyme, the 8-oxoguanine DNA glycosylase, OGG1, to remove oxidized base adducts after oxidative damage (8-Oxoguanine lesions) [55]. Meiotic recombination in oocyte arrested in meiosis I uses HR, while oocytes in meiosis II accumulate transcripts from all the DNA repair pathways to be used after fertilization in the early embryo before transcription starts at the four-cell stage, and the canonical DDR is activated [56,57].

It was recently shown that during sleep, chromatin motion and dynamic in neuronal nuclei match DNA repairs but require a threshold of DNA damage. The general mechanism for this relationship remains to be deciphered. On the contrary, sleep deprivation affects NER, BER, HR repair processes and ERCC1, OGG1, and XRCC1 genes are decreased [58,59].

### 3.2. Cell Cycle Checkpoints

**Figure 2:**
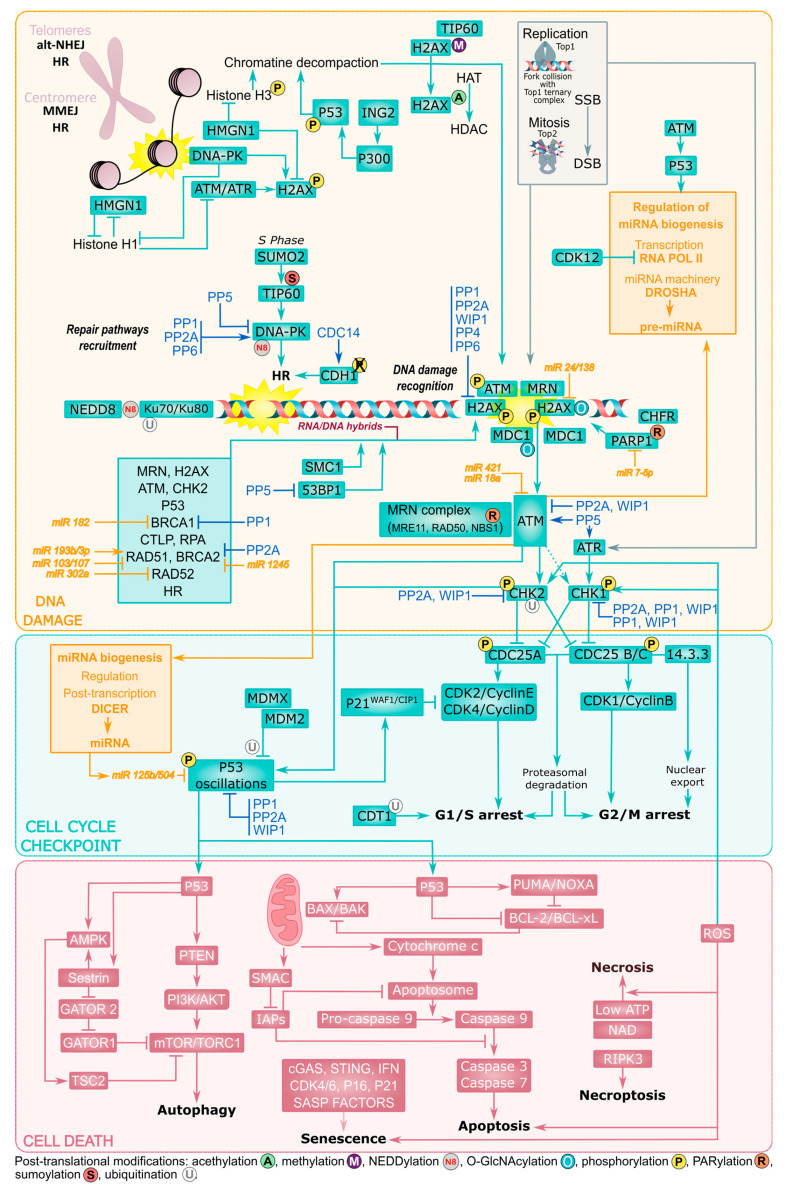
Regulation of the DNA damage signaling network of proteins. In the yellow panel: after DNA damage, induced for example by topoisomerases misregulation, repair pathways are selected, and major sensors/effectors are recruited to the damaged lesions. These recruitments are accompanied by many post-translational modifications of proteins with major impacts on the cell response to DNA damage, e.g., O-GlcNAcylation of H2AX and NDC1 at the DNA damage repair site, ubiquitination and NEDDylation of Ku70/80 from the NHEJ and/or PARylation of NBS1 in the MRN complex from HR, and sumoylation of TIP60 in HR. The chromosomal state and the regions damaged mobilize different repair processes that are facilitated by chromatin decompaction through the action of HMGN1 on histone H3, histones methylation/acetylation, and ING proteins (e.g., ING2) action on phosphorylated P53. Topoisomerases misregulation induces persistent DNA breaks. The cell cycle phase controls the selected DSB repair process: HR acts in S phase. CDH1, a cofactor of APC/C (anaphase-promoting complex/cyclosome, a multi-subunit E3 ubiquitin ligase) involved in cyclin degradation, is activated by CDC14 and by DNA damage in G2, while normally inactive. CDK12 represses transcription by inhibition of RNA POLII (polymerase II). The replication factor CDT1 is proteolyzed in G1 providing a checkpoint control to avoid replication. In the blue panel: early signaling by PI3K (ATM, ATR, and DNA-PK) activates secondary kinases. CHK1 and CHK2 lead to cell cycle checkpoint regulation and arrest the cell cycle, respectively, in G2/M after CDC25C inactivation and G1/S after CDC25A inhibition. Another PI3K, PTEN, triggers cell death. Phosphatases tightly regulate phosphorylation at several levels of the signaling cascades, and counter-balance kinases. Recruited repair effectors, such as ATM, regulate miRNA biogenesis (orange) at the transcriptional and post-transcriptional levels. In return, miRNAs regulate other components of the signaling pathways. RNA/DNA hybrids facilitate the recruitment of DDR effectors to DSB. In the red panel: different cell death processes are triggered by different pathways. P53 oscillations initiate a caspase cleavage cascade and apoptosis, while the inhibition of the mTORC1 complex directs the cell outcome to autophagy. Levels of ATP and NAD dictate necrosis and senescence, and activation of the RIP3 kinase conducts to necroptosis. Senescence is only triggered when P21, P16, and CDK4/6 are activated with IFN-associated inflammatory background and results in the secretion of SASP (senescence-associated secretory phenotype) factors.

Checkpoint pathways arrest the cell cycle progression until DNA integrity is restored by the repair mechanisms [60]. Phosphatidylinositol 3’ kinase-related kinases (ATM, ATR, DNA-PK) phosphorylate multiple substrates. For example, a DSB allows the recruitment and the phosphorylation of the MRN (MRE11-RAD50-NBS1) complex and the ATM activation that further phosphorylates several targets: SMC1 (structural maintenance of chromosomes protein 1) activates the S-phase checkpoints and halts DNA replication, the BRCA proteins involved in HR repair, and NBS1 necessary for the ATM-dependent phosphorylation of CHK1 and CHK2. CHK1 and CHK2 phosphorylate phosphatases CDC25B/C and CDC25A promoting, respectively, a cell cycle arrest in G2/M and G1/S. These arrests involve the binding of CDC25 to 14-3-3 and a self-accelerated proteasomal degradation of CDC25. Therefore, the activation of the cyclin/CDK complexes by dephosphorylation on Thr14 and Tyr15 does not occur. Moreover, the phosphorylation of MDC1 (mediator of DNA damage checkpoint 1) by ATM, activates the S and G2/M cell cycle checkpoints and is facilitated by the phosphorylated histone H2AX (γH2AX). The phosphorylation of P53, by ATM and CHK2, activates P21^WAF1/CIP1^ and Bax genes (BCL2 associated X protein), and, respectively, arrests cells in G1 or triggers apoptosis. Phosphoprotein phosphatases PP1, PP2A, PP2Cδ (WIP1), PP4, and PP6 restrain the response intensity with an action on several targets involved in recognition, signaling or recombination processes. PP5 has a contrasting activity by stimulating ATM signaling and restraining DNA-PK activity [61,62]. WIP1 is necessary for BRCA1 recruitment to the DNA lesion, 53BP1 dephosphorylation, and to promote HR repair [63] (Figure 2).

Cells are sensitive to DNA damage during mitosis and hypersensitive in antephase when they prepare to enter the M phase [64]. Even though the DDR is partially inactivated in mitosis, H2AX is phosphorylated, and the DNA-PK and MRN complexes, MDC1, and the DNA topoisomerase II binding protein 1 (TopBP1) are recruited [45]. After a DSB, mitosis progression is not halted unless a DNA break affects the centromeric or telomeric regions, while a reversible arrest is initiated in the G1 and G2 phases [65].

The cell cycle controls the competition between DSB resection-dependent repair pathways HR, SSA, Alt-NHEJ, and NHEJ. A DSB repair proceeds with a copy of the missing information from the sister chromatid during the S and G2 phases with the phosphorylation of the members of the MRN complex, BRCA1 and CtIP by the ATM kinase, favoring the three resection-dependent DSB repair pathways (HR, alt-NHEJ, or SSA). HR depends on CDK/cyclin and the phosphorylation of multiple substrates, including the MRN complex and CtIP. In the G1 phase, RIF1 and 53BP1 localize to the DSB, inhibit the recruitment of BRCA1, block the DNA end resection pathways, and promote NHEJ. The HR process competes with the NHEJ in interphase. The regulation of DNA-PK during the cell cycle determines the choice of the DNA repair pathway. After a DSB, in the G1 phase of the cell cycle, DNA-PK autophosphorylation on Ser2056 and activation is facilitated by its interaction with TIP60 histone acetyltransferase (HAT), and the repair process executes a NHEJ. In the S phase, the SUMO2 (small ubiquitin-related modifier 2) modification of TIP60 mediated by the PISA4-E3-ligase blocks the TIP60-induced DNA-PK activation, and HR repair is favored [58].

Cell cycle chromatin modifications regulate the coupling between DNA replication and mitosis. The initiation of DNA replication is controlled by CDT1 licensing factors. CDT1 accumulates from the M to the G1 phase and is degraded in the S phase (after poly-ubiquitination by Cullin-4-ring E3 ubiquitin ligase) to ensure a correct onset of DNA replication and prevent re-replication. In response to DNA damage, CDT1 is proteolyzed in G1 providing a checkpoint control [66].

### 3.3. Cell Death and P53 Signaling

The cellular outcome after the kinases cascades depends on the types and the severity of the DNA damage. Cells with excessive DNA damage or unrepairable DNA undergo a programmed cell death involving a P53-dependent mechanism such as apoptosis, or other types of cell death that do not rely on caspase-dependent activation (Figure 2).

P53 accumulates after a negative feedback loop involving an MDM2 (Murine double minute 2) inhibitor with a ubiquitin ligase activity and WIP1 phosphatase to induce cellular senescence or apoptosis. The P53 level oscillates and the oscillation numbers directly relate to the DSB quantities and to the cell death outcome that depends on the genetic context of the damaged cells [67]. A failure in these oscillations allows the cell to escape the G1 arrest. When P53 is mutated in a high-grade serous carcinoma, their reliance on the G2/M checkpoint is increased [68]. P53 maintains a cell arrest, initiates senescence through the activation of P21^WAF1/CIP1^, inhibits cyclin-dependent kinases, and the binding of retinoblastoma protein to E2F transcription factor to regulate the G1/S transition. P53 phosphorylation on Ser46 is specifically linked to the pro-apoptotic expression of PUMA and NOXA proteins, the release of mitochondrial cytochrome C, the cleavage of caspases, and apoptosis [69,70]. An exception is the mature spermatozoa that do not complete apoptosis as their nucleus and mitochondria are localized in different compartments [71]. Autophagy is mediated by ATM and nuclear P53, which activates the transcription of PTEN (phosphatase and tensin homolog), AMPK, and Sestrin that engages TSC2 (Tuberous Sclerosis Complex 2) and AKT to inhibit mTORC1. The inhibition of the DNA-PK or ATR/CHK1/RhoB-mediated lysosomal recruitment of a TSC complex also results in autophagy [72]. mTORC1 connects to the cell cycle checkpoint through the GATOR1 complex component NPRL2, which arrests the cell cycle in G1/S in P53 positive cells and in S or G2/M in P53 negative cells. mTORC1 also negatively controls ATM via S6K1/2 signaling by up-regulating two miRNAs (miR-18a and miR-421) targeting ATM mRNA [73,74]. The cellular depletion of ATP and NAD causes oxidative stress, activation of the RIPK3 (receptor-interacting protein kinase 3), and necroptosis [75]. In dividing cells, DNA lesions interfere with DNA synthesis (replication forks stalling) and the incompletely replicated DNA causes mitotic catastrophe when cells try to break through G2-arrest into the M phase [76].

Another member of the P53 family is involved in oocytes facing cumulative DNA damage when arrested in the prophase of the first meiosis division for many years in ovaries [77]. Prophase 1 oocytes in primordial follicles are unable to respond immediately to DNA damage, while all components necessary for the DNA repair including direct lesion reversal, NER, BER, MMR, HR, and NHEJ processes are present [56,78]. They depend on a G2/M DDR and P63, a member of the P53 family, activated by ATM and CHK1. During their meiotic arrest, DNA-damaged oocytes undergo a specific P63-dependent apoptotic death [79] only for severe DNA damage [80]. Later, in metaphase I and II oocytes of growing follicles, the DNA damage-induced arrest is overridden by the spindle assembly checkpoint (SAC) [81,82].

### 3.4. Chromatin State and Chromosomal Damage Location

The native chromatin state (heterochromatin, just transcribed DNA) and the type of damage (blunt end, overhang, adducts) influence the choice and the kinetic of the repair processes.

Chromatin compaction, such as heterochromatin, limits the accessibility of DNA damage response proteins to the DNA damage sites, making these regions difficult to repair. Chromatin local relaxation by histone H1 and high mobility group proteins (HMGs) is an early event of the DDR. HMGN1 binds to nucleosomes and reduces histone H3 phosphorylation on Ser10, a necessary site to maintain the chromatin condensed state in mitosis. TIP60 acetylates histone H2AX, a prerequisite to both its phosphorylation and ubiquitination. The phosphorylation of H2AX is mediated by ATM, ATR, or DNA-PK on the nucleosomes flanking accidental DSBs but also in natural processes of meiosis and the immune system during DNA recombination. This is an indirect effect of ING (inhibitor of growth) proteins, as ING2 on P53 is essential for the chromatin-induced decompaction allowing accessibility of the repair proteins to DNA damage sites and providing the necessary signal to translocate ATM and the MRN complex to the damaged DNA site [83]. A DSB in actively transcribed chromatin is preferentially halted and repaired by HR and end resection in G2, as they are absent in G1 [84,85]. Inactive lamina heterochromatin prefers alt-EJ [86]. Nuclear position dictates the DNA repair pathway choice. Transcriptionally active chromatin recruits HR at DNA DSBs. Open chromatin (in promoters and enhancers) is more permissive for end resection [87]. Specific chromatin locations have repair pathway preferences. Damage in the telomeres tends to inhibit NHEJ and to select alt-NHEJ or HR repair processes; centrosomes, the microtubules-organizing centers, rely on HR or MMEJ (resection mechanisms) [45,88].

### 3.5. Post-Translational Modifications

Proteins from the DDR are controlled by post-translational modifications, revealing a complex organizational response. Post-translational modifications modulate the chromatin structure and provide docking sites to recruit and accumulate DDR repair proteins at the DNA damage site. Phosphorylation is one of the central proteins post-translational modification and also the most studied in the initiation and the execution of the DDR response. Many different DDR proteins involved in checkpoint control and DNA repair are regulated by a cycle of phosphorylation/dephosphorylation and by the presence of DDR-kinase/phosphatase molecular switches. The kinases ATM/ATR/DNA-PK and CHK1/2 cascades are central networks in DDR activation.

Following DNA damage, several phosphatases participate in the DNA damage checkpoint activation and regulate several phosphorylations at multiple levels by dephosphorylating sensor kinases, ATM, ATR, and DNA-PK, transducer kinases CHK1/2, histones H2AX and H3, and effectors such as P53 and MDM2 [89]. PP1, PP2A, WIP1, and PP5 dephosphorylate ATM, allowing its recruitment to damaged sites [89,90]. PP5 is required for the ATR-dependent phosphorylation of CHK1 [91]. PP2A, PP5, and PP6 dephosphorylate DNA-PK on multiple sites to enhance its activity [92,93,94]. WIP1 dephosphorylates CHK1, promoting its release from chromatin and an increase in its kinase activity [95], and antagonizes CHK2 [96]. PP2A, PP4, PP6, and WIP1 dephosphorylate γH2AX, and PP1 dephosphorylates H3, leading to the transcriptional repression of cell cycle-regulated genes [62]. PP1 associates to PNUTS (protein phosphatase 1 nuclear-targeting subunit) [97] and WIP1 dephosphorylates and inactivates P53 [98]. PP1 dephosphorylates and positively regulates BRCA1 [99]. PP5 dephosphorylates 53BP1, leading to its release from DNA damage sites, while normally recruited to DNA damage to coordinate DDR factors localization and activation [100]. CDC14 triggers nuclear accumulation and the activation of YEN1 at anaphase to resolve recombination repair intermediates [101].

Distinct ways of regulation are used by phosphatases to regulate upstream DDR signals. WIP1 dampens the DDR with a feedback circuit, while PP2A provides fine-tuning and acts with P53 in a negative feedback loop [102] that is necessary for efficient DNA repair and post-repair restart of cell division [103]. PP2A keeps CHK2 inactive in normal conditions but, following DNA damage, CHK2 is released and followed by a late reconstitution of the PP2A/CHK2 interaction, increasing the threshold necessary for CHK2 activation [104]. Phosphatases are submitted to the DDR kinetic regulation working on different repair substrates during different time periods after DNA damage. Indeed, PP4 dephosphorylates and maintains RPA2 in a hypo-phosphorylated form immediately after DNA damage to avoid competition for ssDNA binding with other factors such as RPA1 [61,105,106].

Several other covalent modifications including ubiquitylation, sumoylation, poly-ADP-ribosylation (PARylation), neddylation, acetylation, and methylation facilitate protein recruitments to the damaged DNA [47]. SUMO and ubiquitin conjugates compete for lysine residues in target proteins [107]. The poly ADP-ribose polymerases (PARPs) modify proteins by linear or branched chains of ADP-ribose units originating from NAD rapidly after the induction of DNA breaks. More than 90% of PAR addition is mediated by PARP1. PARylated proteins are recruited through their PAR binding site. PARylation is capable of prime ubiquitination at the site of DNA lesions for mediators such as CHFR ubiquitin E3 ligases [108]. Neddylation is the link of the ubiquitin-like protein NEDD8 through its glycine carboxy-terminal to a lysine of the targeted protein, such as Ku70/80 and DNA-PK [109,110]. HATs (histone acetyltransferases) and HDACs (deacetylases) catalyze the addition or removal of acetyl groups on lysine residues, altering the chromatin structure and providing binding signals for proteins with recognition domains, to mediate key DDR activities [111]. Histone and non-histone lysine methylation can alter the protein’s interaction with chromatin and recognition by reader proteins [112]. Similar to phosphorylation, the O-linked N-acetylglucosamine linkage (O-GlcNAcylation) is realized on serine or threonine residues [113]. OGT (O-GlcNAc transferase) relocates to a DNA-damaged site, allowing for the O-GlcNAcylation of the H2AX histone and MDC1 and restrains the DDR to a specifically damaged site. The inhibition of OGT also delays DSB repair, reduces cell proliferation, and increases cell senescence [114].

### 3.6. ncRNAs as Emerging Regulators

Local RNA synthesis or processing, and non-coding RNA have emerged as novel pathways in the regulation of DDR. Transcription is globally impaired in response to DNA damage, with ATM/DNA-PK triggering the ubiquitination of RNAPII by NEDD4 ligase and proteasomal degradation but opening chromatin at the site of the DSB locally to allow RNA synthesis [115,116]. Moreover, DNA damage affects mRNA splicing and stability, and mRNA splicing affects DDR. Serine/arginine-rich splicing factors (SRSFs) and heterogeneous nuclear ribonucleoproteins (hnRNP) are phosphorylated by ATM/ATR. HnRNP is co-recruited with the BCRCA1/PALB2/BRCA2 complex that further recruits Rad51 and regulates proper splicing at the DNA damaged site [117].

R-loops are three-stranded structures formed by an RNA/DNA hybrid and a displaced single-stranded DNA that plays multiple roles in gene expression, DNA replication, immunoglobulin CSR, and DNA repair [118]. In normal conditions, several mechanisms prevent the deleterious effects of an accumulation of deregulated R-loops. The THO/TREX (TRanscription-EXport) and TREX-2 mRNA export complexes function in proper packaging of mRNA into export-competent mRNPs, minimizing the possibility for a nascent mRNA to re-hybridize with the strand of transcribed DNA [19]. DNA topoisomerases relax DNA-negative supercoils and SRSF1 packs nascent mRNA into ribonucleoprotein complexes in a topoisomerase 1-dependent manner [119,120]. An SR protein, the alternative splicing factor/splicing factor 2 (ASF/SF2), regulates the formation of R-loops [121]. However, the accumulation of diverse RNA-containing structures, including RNA/DNA hybrids, generates DNA damage. It has been accepted that R-loops present a block for the replication fork progression, and constitute a major cause of replication stress that gives rise to genetic instability and chromosome fragility [122,123]. R-loop-mediated genomic instability is caused by the impairment of replication fork progression [124] and a deficiency in the molecular factors responsible for R-loops prevention. On the contrary, other studies have shown that RNA/DNA hybrids are involved in DSB repair processes in HR or NHEJ [125,126,127]. Hybrids protect DNA 3’overhangs, preventing an excess of resection [125,128], and contribute to the recruitment of DNA repair factors such as BRCA1 [129], 53BP1 [130], RPA [131], and FA pathway proteins, including FANCD2, FANCA, and FANCM. Senataxin, in complex with BRCA1, is recruited to R-loop-rich termination regions [129]. The R-loop-driven ATR pathway acts at centromeres to promote faithful chromosome segregation [132].

DNA damage induces the production and the control of a variety of non-coding RNA (ncRNA), as follows: microRNA (miRNA), and small or long ncRNA (s/lncRNA). P53 induces lncRNA expression that controls gene expression, cell cycle, and apoptosis; DSB-induced small RNA contributes to HDR; ATM, BRCA1, and P53 regulate miRNA expression; DROSHA/DICER-dependent RNA are involved in the formation of DNA repair foci [133]. A class of sncRNA, the DNA damage response RNAs, generated in a DROSHA/DICER-dependent manner are required to recruit secondary DDR factors at the chromosomal and the telomeres DSBs [134]. Several DNA damage-induced effectors control the transcription processes of non-coding RNA by the regulation of RNA interference factors DROSHA/DICER, and RNA polymerase II [115]. On the reverse, ncRNA plays various roles in damage signaling and repair by a regulation of the quantity of DNA repair proteins, by guiding or by providing a template for the DNA reparation (Figure 2). Nearly all primary DDR-induced effectors are regulated by miRNA and interact through their RNA-binding motifs in response to DSBs [126]. MiRNAs regulate, for example, H2AX (miRNA 24 and 138 [135,136]), PARP1 (miRNA-7-5p [137]), ATM (miRNA 18a and 421 [138]), ATM and DNA-PK (miRNA 101 [139]), BRCA1 (miRNA 182 [140]), RAD51 (miRNA 193b, 103 and 107 [141]), BRCA2 (miRNA 1245 [142]), RAD52 (miRNA 302a [143]), and P53 (miRNA 125b and 504 [144,145]). Actual models propose that ncRNAs facilitate DNA repair, either by the hybridization of sncRNA to broken DNA via DDX1 and DHX9 (dead-box helicases) to form specific sequences propagating repair pathways or by the hybridization of lncRNA via RAD52-strand invasion, generating a template for high-fidelity repair [146,147].

LncRNA has proven regulatory modulation of DDR. They attenuate ATM activation and HR repair, sensitizing genotoxic treatment in cancers [53]. MiRNAs and lncRNAs cooperate with P21^WAF1/CIP1^ (CDK inhibitor) to affect protein expression [148]. This generates cross-signatures in glioma and could serve malignancy diagnosis and prognosis [149]. In actively transcribed regions, lncRNA serve as template for NHEJ-mediated DSB repair to allow for the faithful transfer of missing information [150].

## 4. DDR Alterations and Associated Diseases

**Figure 3:**
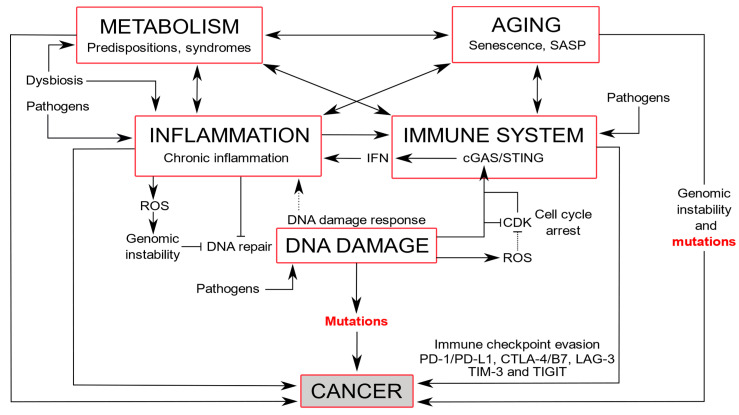
DNA damage induces inflammation and immunity that connect to metabolism and aging, with the genesis of mutations leading to cancer formation. The DNA damage response is activated upon DNA damage, arresting the cell cycle by CDK inhibition, and triggering an inflammatory response. cGAS, a cytosolic DNA sensor, triggers an innate immune response that activates STING. In mitosis, the CDK1-cyclin B complex phosphorylates and inhibits cGAS, but upon mitotic exit, dephosphorylation of cGAS by PP1 enables DNA cytoplasmic sensing [163]. cGAS connects DNA damage to the immune system, and subsequent inflammation by the production of interferon (IFN) and cytokines, but also to senescence and cancer [164]. Chronic inflammation can lead to an impairment of the immune system and generate ROS that counteracts DNA repair mechanisms [165]. Immunity triggers an inflammatory process that increases blood pressure, stimulating organ and metabolism damage [166,167]. Metabolism shifts with increased oxygen consumption and the generation of reactive nitrogen and oxygen intermediates are associated with inflammatory and immune responses [168]. Dysbiosis produces inflammation and metabolism syndromes that contribute to senescence. Pathogens can induce DNA damage and trigger immune and inflammatory responses. Aging is associated with adaptative immune and inflammatory responses and cumulative DNA damage and genomic instability that increase mutations [169,170]. Cancer cells evade detection by the immune system using immune checkpoints PD-1/PD-L1 (programmed cell death protein 1/programmed cell death ligand 1) and CTLA-4 (anti-cytotoxic T lymphocyte-associated antigen-4), LAG-3 (lymphocyte activation gene-3), TIM-3 (T cell immunoglobulin and mucin domain containing-3), and TIGIT (T cell immunoglobulin and ITIM domain) [171].

The alterations of many DDR members are associated with a range of diverse diseases, including immunodeficiency, inflammation, neurodegeneration, premature aging, cardiovascular or metabolic diseases, and cancers. However, there are reciprocal interactions between DNA damaged/repairing responses. For example, under viral infection, immunity directly relates to inflammation that underlies premature aging, and a high risk of cancer (Figure 3).

### 4.1. Immune Diseases and Inflammation

Immunodeficiencies are associated with DNA repair defects in the process of V(D)J genes and immunoglobulin class-switch recombinations. In immunoglobulin class switch recombination, a DSB is induced by the activation of a cytidine deaminase before broken ends are repaired by NHEJ or MMR. Any deficiency in one of the effectors of these repair complexes impairs the generation of IgG diversity to fight foreign antigens (acquired immunity), and leads to immunodeficiency at various degrees or hyper-IgM syndrome [151].

Different components of the DDR activate the innate immune system through the expression of ligands for the immune cell receptors and promote the production of proinflammatory mediators [152,153]. ATM, ATR, and CHK1 upregulate the formation of a ligand for the NKG2D immune receptor triggered by the stalled DNA replication fork [154].

The association of pre-existing DNA-repair defects (e.g., PARP1 or XRCC1/4) with DNA-damaging antibodies is highly genotoxic. In autoimmune diseases, such as systemic lupus erythematosus, anti-DNA autoantibodies circulate in the blood, cross cell membranes through mechanisms independent of their Fc region, and translocate in the nucleus to inhibit DNA repair and even damage DNA [155,156,157].

DNA damage effectors detect aberrant DNA structures in the nucleus and the cytoplasm, leading to immune signaling activation. For instance, Ku70 and DNA-PK, which are involved in the NHEJ, act as pathogenic DNA-recognition receptors in the cytoplasm and activate proinflammatory interferon, respectively, directly by IRF3 (IFN regulatory factor 3) or indirectly via STING (stimulator of interferon genes) signaling [158]. PARP1 upon genotoxic stress or the ATM activation of IκB kinases in DSBs both induce the translocation of NF-κB into the nucleus and promote the proinflammatory interferon and cytokine mediator’s production. A loss of ATM impairs the innate inflammasome-dependent antibacterial immunity due to oxidative stress [159].

Moreover, an infection causes inflammation and enhances the production of ROS and oxidative DNA damage, creating a positive feedback loop that promotes genomic instability, cellular transformation, and carcinogenesis [160]. In the innate immune response, the cGAS–cGAMP–STING pathway senses cytoplasmic DNA, including microbial DNA or DNA released by DNA damage, and allows the production of more interferon and cytokines, the hallmarks of inflammation [161]. This pathway is also involved in the detection of DNA damage induced by autoantibodies in the systemic lupus erythematosus and directly connects this disease to inflammation by chronic interferon release [162].

### 4.2. Neurodegeneration and Premature Aging

Neuronal dysfunction and neurodegenerative diseases occur with higher frequency when a persistent DDR is set up in neurons in association with an enhanced cholesterol biosynthesis and impaired Wnt or insulin (GSK3β increase) pathways [172]. In Huntington’s, Parkinson’s, and Alzheimer’s diseases, PARP1 is increased. In Huntington’s disease, NHEJ is defective and elevated PARP1 leads to increased AMPK activation, decreasing the CREB (C-AMP response element-binding protein) transcription factor necessary for the survival factor BNFD (brain-derived neurotrophic factor) and, finally, promotes necrotic cell death. In Parkinson’s disease, increased levels of PARP1 accelerates the fibrillization and cytotoxicity of α-synuclein, which further activates ATM, H2AX, 53BP1, and NHEJ. In Alzheimer’s disease, P53 stabilized by PARylation causes the upregulation of pro-apoptotic protein [173]. Moreover, Tau hyperphosphorylation, and abundant neurofibrillaries downregulate DNA damage/repair pathways with abnormal BRCA1 location and cell cycle re-entry inhibition [174].

Due to germline mutations in relevant genes, specific syndromes linked to DNA repair defects are hereditary. Distinct chromosomal instability disorders ataxia telangiectasia, Bloom syndrome, Cockayne syndrome, Fanconi anemia, Nijmegen breakage syndrome, trichothiodystrophy, Werner syndrome, and xeroderma pigmentosum display hypersensitive cells to particular genotoxic compounds. They also share clinical features of growth retardation, neurological disorders, premature aging, and skin alterations [175]. In ataxia telangiectasia, ataxia with oculomotor apraxia 1 and 2, and spinocerebellar ataxia with axonal neuropathy 1, the cerebellum is particularly affected. Neurodegeneration in xeroderma pigmentosum, Cockayne syndrome, and trichothiodystrophy is associated with NER deficiency [176]. These progeroid syndromes feature some aspects associated with physiological aging at an early age.

### 4.3. Aging

Several lines of evidence indicate that the aging process is an association of DNA damage accumulation due to decreased efficiency in repair processes, mitochondrial dysfunction, metabolism alteration, free radical accumulation, and telomeres shortening [177,178,179]. The persistence of DNA modifications affects DNA polymerization, resulting in the collapse of the replication fork, an arrest in transcription, and the genesis of mutagenic templates during the synthesis of nucleic acid leading to cellular aging [180]. Aged cells accumulate extranuclear DNA, which triggers inflammation initiated by cGAS-STING and senescence [181]. Senescence is associated with the inhibition of mitophagy [182]. The loss of sirtuin NAD-deacetylase activity due to lower NAD+ levels inhibits mitophagy and results in persistent dysfunctional mitochondria and aging [183]. Moreover, proteins from the DDR lack deacetylation, such as ATM, XA, WRN helicase, and Ku70, impairing the repair processes [184].

DNA repair proteins become less efficient with age, especially in germline cells where DNA damage accumulates within the genome [185]. After remaining in the prophase of the first meiotic division for years, the quality of oocytes declines with age. An increasing number of primordial follicles accumulate severe DSBs and are submitted to apoptosis that diminishes the ovarian reserve [186]. In the mid-30s, in the remaining oocytes, aneuploidy increases due to a decreased efficiency in BRCA1 and ATM that is necessary for the regulation of sister chromatid cohesion by a control of the ATM-regulated SMC1 cohesin complex [185] and ROS vulnerability [187]. During the menopause, DNA damage accumulates in oocyte due to the reduction in DNA repair capacities, such as a decrease in BRCA1 [185]. However, human oocytes submitted to potent DNA damaging agents applied chronically at high concentrations extrude their polar body and complete meiosis I, and have a higher acceptance of DNA-damage compared to other species [80].

### 4.4. Cardiovascular and Metabolic Diseases

DNA damage is a hallmark in cardiovascular and metabolism disorders. In failing hearts and cardiovascular diseases, oxidative stress (redox imbalance) and DNA damage are enhanced [123,188]. The BRCA1-deficient cardiomyocytes that have an impaired DSB repair activate p53-mediated proapoptotic signaling, whereas P53 deletion rescues BRCA1-deficient mice from cardiac failure. In addition, ischemia induces DSBs and upregulates BRCA1 expression in adult and fetal cardiac tissues [189]. An SSB was shown to cause heart failure by activating the DDR and increasing the expression of inflammatory cytokines via NF-κB signaling. Heart failure was more severe in the mice lacking XRCC1, but was inhibited by the genetic deletion of ATM [190].

In atherosclerosis, where the atherosclerotic plaque instability is associated with inflammatory cells, multiple DNA damage markers are present, including early induced DNA repair enzymes and the phosphorylated forms of ATM and H2AX [191]. The vascular smooth muscle cells show a defective repair of oxidative DNA damage, due to the increased degradation of the BER enzyme, OGG1 [192]. In atherosclerotic cardiovascular diseases such as the Werner or Hutchinson-Gilford progeria syndromes, DSB repairs are defective [193]. Both syndromes are also, respectively, associated with diabetes and lipodystrophy symptoms. The metabolic changes in type 1 and type 2 diabetes increase the levels of genotoxic ROS and dicarbonyls that further form glycation adducts and reduce DNA repair [194].

Chronic diabetic type 2 β-cells have a greater extended DSB [195]. A compromised DNA repair is responsible for diabetes-associated fibrosis in various organs [194].

In obesity, oxidative stress and inflammation induce DNA damage, inhibit DNA repair, alter gene expression to set up metabolism disturbances, and further promote resistance to apoptosis and predispose to cancer [196]. Adipocytes’ metabolic and secretory functions are strongly altered by DNA damage [197]. On the contrary, weight loss may prevent obesity and other adverse health effects due to a reduction in DNA damage with a decrease in pro-inflammatory cytokines and insulin levels [198].

### 4.5. Cancers

Various mechanisms related to DNA damage including dysregulation of the DDR pathways and a high level of replication stress are responsible for genomic instability, and linked to pretumor and tumor formation [44]. Aberrant copy numbers of genes correlate with the expression levels of mRNA [199]. Various primary cancers also display mitochondrial DNA mutations involved in ROS production, apoptosis inhibition, DNA repair anomalies, and cell cycle misregulation in the mitotic spindle and the G2/M checkpoint [200,201].

More than 450 proteins from the DDR [2] are related to familial cancer predispositions through diverse somatic mutations or inactivating germline mutations (Table 1). BRCA1 and BRCA2 are associated with a family history of breast and ovarian cancers. ATM is associated with a family history of breast and ovarian cancers with 1.7 times higher risk [202,203]. MSH2/6 is associated with a family history of colon/endometrial cancers. The “ICGC/TCGA Pan-Cancer Analysis of Whole Genomes Project Consortium 2020” has shown that somatic mutations of DDR-related genes were found in a third of cancers [204]. Multiple processes generate somatic mutations in the genome and determine a mutational signature for each type of cancer. These studies have identified defective components from the DNA damage repair, replication, the DNA-maintenance processes [204], and associated mutations, for instance, CDK12 (transcriptional modulator of DDR genes and mRNA processing) with DNA duplicated tandem stretches, or truncated variants of the MMR enzyme MBD4 (methyl-CpG-binding domain protein 4) with a mutational signature of CpG sites [205].

Moreover, interconnections are present between chronic inflammation or infection and generate genotoxic stress and tumor initiation/progression [206,207]. The risk of colorectal cancer is underlined by faster DNA damage in inflammatory bowel disease [208]. Tumor cell proliferation is also linked to the remodeling of the immune microenvironment through the deregulation of rhythmic expression of cyclin genes and the P21^WAF/CIP1^ cell cycle inhibitor when the circadian rhythm is disrupted [209]. A virus such as the human papillomavirus reinitiates DNA replication by abrogating the cell cycle checkpoint proteins P53 and Rb (retinoblastoma protein), leading to an unscheduled S phase entry, followed by replication stress, DDR, and carcinogenesis [210].

**Table 1:** DDR-proteins mutations associated to various cancers.

DDR Proteins	DDR Signaling Pathways	Protein Activity/Function	Cancer-Associated Mutations *
ATM	Cell cycle regulators	Kinases	Breast, colon, endometrial, leukemia, lung, lymphoma, pancreatic, prostate
ATR			Breast, colon, endometrial, gastric, lung, lymphoma
CHK2	Cell cycle	Phosphatases	Bladder, breast, colon, endometrial, lung
APC-C/CDH1			Breast, gastric, lung
PTEN	Cell death	Phosphatase	Breast, endometrial, gastric
P53		Transcription factor	Found in 39.52% of all cancers *(AACR)*
**DNA repair proteins**	**DNA repair pathways**		
Ku70/Ku80	NHEJ	Helicases	Breast, colorectal, lung, melanoma
DNA-PK		Kinase	Breast, colon, glioma, oesophagal, lung
NBS1	HR	MRN complex	Breast, colon, esophageal, head and neck, hepatoma, liver, lung, lymphoma, prostate
MRE11			Breast
RAD50			Colon, gastrointestinal, lung
BRCA1		E3 ubiquitin-ligase	Breast, colon, gastrointestinal, haematological, lung, melanoma, ovarian, pancreatic, prostate
BRCA2		RAD51 binding to DNA	
PALB2/FANCN		Recruitment of BRCA2 and RAD51	Breast, colon, head and neck, lung, ovarian
FANCA, FANCB		FA repair complex	Breast, colon, leukemia, liver, lung
FANCO/RAD51C		DNA-dependent ATPase	Breast, colon, lung, ovarian, prostate
RAD51D			Breast, lung, ovarian
BLM		Helicases	Breast, colon, endometrial, leukemia, lung, lymphoma, melanoma
WRN			Colorectal, endometrial, lung, melanoma, pancreatic, thyroid
ERCC1	NER	Nuclease	Colorectal, glioma, lung, skin
XPA, XPC		Scaffold protein	Bladder, colon, lung, skin
XPD/ERCC2		Helicase	
XPG/ERCC5		Endonuclease	
OGG1	BER	Glycosylase	Breast, lung, renal
PARP1		ADP-ribosyltransferase	Breast, colon, endometrial, lung
XRCC1		Scaffold protein	Non-small cell lung
MLH1	MMR	ATPase	Brain, breast, colorectal, endometrial, hepatobiliary, lung, ovarian, pancreatic, skin, stomach, upper urinary
MSH2, MSH6		Scaffold protein	
MGMT	DR	Methyltransferase	Gliomas

Notes for Table 1: ATM, ATR, CHK1/2, and APC/C-CDH1 are involved in cell cycle regulation; PTEN and P53 in programmed cell death. In NHEJ, the ATP dependent helicases Ku70 and Ku80 form a heterocomplex with DNA-ends, and Ku80 C terminus recruits DNA-PK, a phosphatidylinositol 3-kinase related serine/threonine kinase [211]. In HR, two NBS1 subunits (phosphopeptide-binding Nijmegen breakage syndrome protein 1) are associated with two MRE11 subunits (meiotic recombination 11 homolog 1), and two ATP-binding cassette (ABC)-ATPase (RAD50) to compose the MRN complex [212]. BRCA1, an E3 ubiquitin ligase, and BRCA2 facilitate response to DNA damage. PALB2/FANCN (partner and localizer of BRCA2) has a critical role through the recruitment of BRCA2 and RAD51 to DNA breaks [213]. FANCA and FANCB are associated with other FANC (Fanconi Anaemia) and FAAP (FA-associated proteins) (FANCC, FANCE, FANCF, FANCG, FANCL, FANCM and FAAP20, FAAP24, and FAAP100). They form the FA core complex carrying an E3 ligase activity to monoubiquitinate FANCI and FANCD2 [214], and initiate DNA repair by forming a platform to recruit additional proteins. RAD51C forms distinct complexes, one with a related DNA-dependent ATPase paralog, RAD51D, forming the BCDX2 complex (RAD51B-RAD51C-RAD51D-XRCC2), and one with the CX3 complex (RAD51C-XRCC3) [215]. BLM (Bloom syndrome protein) and WRN (Werner syndrome ATP-dependent helicase) are members of the RecQ helicase family. WRN also displays a 3′ to 5′ exonuclease activity [216]. In NER, ERCC1 has a nuclease activity involved in DNA excision repair. XPD/ERCC2 is a 5′–3′ DNA helicase, XPG/ERCC5 an endonuclease involved in DNA excision repair, and XPA (Xeroderma pigmentosum complementation group A) a zing finger protein involved in nucleotide excision repair [217]. In BER, OGG1 is a 8-Oxoguanine glycosylase and the primary enzyme of the process [218]. PARP1 the poly(ADP-ribose) polymerase 1 binds to damaged chromatin and recruits XRCC1 (X-ray repair cross-complementing protein 1) that interacts with DNA ligase III acting as a scaffold protein [219]. MMR is initiated by MutS α (MSH2–MSH6) or MutS β (MSH2–MSH3) binding to a dsDNA mismatch, before MLH MutL alpha (MLH1-PMS2) is recruited to the heteroduplex. MSH2 seems to act as a scaffold, MSH6 has a DNA-dependent ATPase activity, and MLH1 has a nucleotide-binding capability [220]. The O-6-méthylguanine-DNA-méthyltransférase, MGMT, carries out direct repair (DR) of alkylated DNA *: [204,221].

## 5. DDR Targets in Anticancer Therapies

### 5.1. Targeting DDR Proteins to Induce Deficiencies

**Figure 4:**
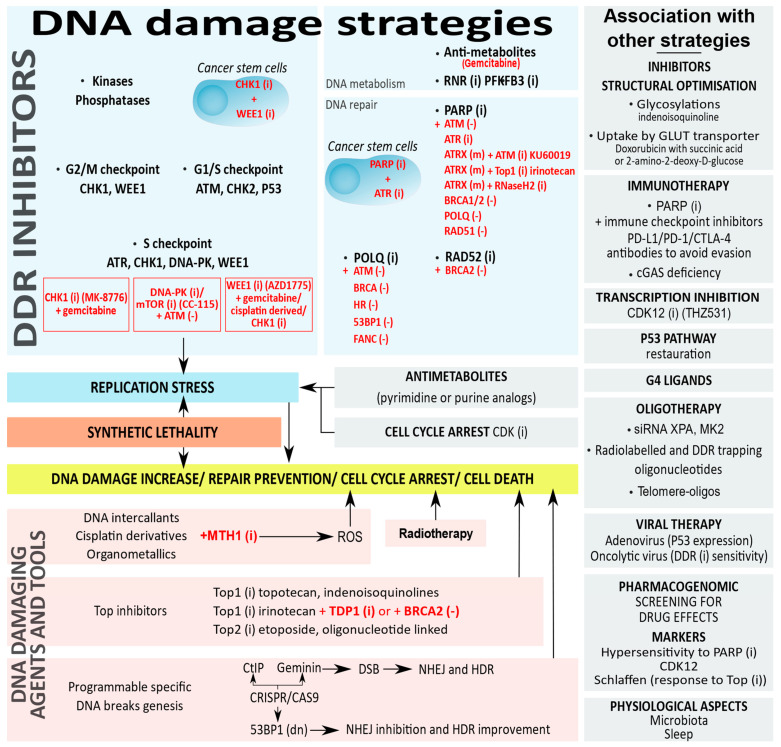
DNA damage strategies in cancer chemotherapies and possible combined therapies. Synthetic lethality is in red. Deficiency (-), dominant negative (dn), inhibitors (i), mutations (m).

Several categories of DDR proteins are targeted in anticancer monotherapies to elicit cell death [222,223] (Figure 4). DNA damage signaling inhibitors target kinases: ATM and DNA-PK are among the first activated kinases upon a DSB and, respectively, execute checkpoint signaling or DNA repair, while ATR is recruited by replication stress to facilitate fork stabilization and restart. ATR and ATM, respectively, activate the kinases CHK1 and CHK2, and WEE1. ATM and CHK2 are G1/S checkpoint DDR protein targets, ATR, CHK1, DNA-PK, and WEE1 are S phase regulators, and CHK1 and WEE1 are G2/M checkpoint regulators. Clinical trials are still currently running for this category of inhibitors, such as the trial in phase I for an oral ATR inhibitor BAY-1895344 against an advanced solid tumor [222,224,225].

Another category of inhibitors targets proteins involved in DNA repair such as RAD51 and POLQ, respectively from the HR and MMEJ processes, or PARP and MTH1 [226,227,228,229]. The discovery of PARP inhibitors was a milestone for anticancer chemotherapies directed against DDR proteins. Initially evaluated in patients with BRCA-mutated breast tumors, the inhibition of PARP eliminates its trapping on DNA and elicits death [230].

A third class of inhibitors is raised against proteins involved in DNA metabolism such as inhibitors for RNR (ribonucleotide reductase) that catalyzes a rate-limiting step in the production of deoxyribonucleotides and PFKFB3 (6-phosphofructo-2-kinase/fructose-2,6-biphosphatase 3) that converts fruc-tose-6-phosphate to indirectly stimulate glycolysis [231,232].

A fourth category of inhibitors is directed against chromatin regulation. DNA methylation or histone methylation/acetylation are epigenetic modifications that affect DNA repair ability. Histone deacetylase (HDAC) inhibitors lower DNA damage repair proteins from the NHEJ and the HR through the inhibition of ATM and MRN [233,234]. DNA methyltransferase 1 (DNMT1) inhibition induces the depletion of multiple repair protein in the MMR [235].

### 5.2. Targeting the DDR Protein Network with Synthetic Lethality

By exploiting the defects in DNA repair, several categories of DDR proteins are targeted to take advantage of the concept of synthetic lethality and induce cell death. Cells usually repair DNA damage by switching from one alternative repair mechanism to another if a repair pathway is defective. Moreover, a loss of function targeting several DDR pathways renders these cells less susceptible to survival. The concept of synthetic lethality used to target DDR pathways occurs when deficiencies in independent genes are tolerated but when the combinations are lethal [230,236,237].

Several DDR inhibitors are investigated in ongoing clinical trials and the choice of the combination of inhibitors depends on the characteristic of the tumor [238]. Synthetic lethal vulnerabilities are exploited therapeutically by combining inhibitors against a tumor deficiency in the DDR protein: CHK1 inhibitor (MK-8776) is used with gemcitabine (antimetabolite) in solid tumors [239], WEE1 Inhibitor (AZD-1775) with gemcitabine, cisplatin, or carboplatin (DNA damaging agents) in advanced solid tumors [240] or in combination with a checkpoint kinase CHK1 inhibitor (PF-0047736) in acute lymphoblastic leukemia [241], and a DNA-PK and mTOR dual inhibitor (CC-115) in hematopoietic and solid cancer cells [242].

Synthetic lethality is explored with PARP inhibitors in several cancers such as ATM-deficient tumors from breast and ovaries, aggressive prostate cancers in combination with ATR inhibitors [243,244], and high-risk neuroblastomas under ATR inhibition [245]. POLQ deficiency is synthetically lethal in combination with PARP inhibitors and associated with mutations of ATM, BRCA, Ku, 53BP1, and FA (Fanconi anaemia) [226,229,246]. Other synthetic lethalities include the ATM inhibitor, KU60019, or Top1 inhibitor, irinotecan, in ATRX-deleted neuroblastoma [247]. The application of synthetic lethality also depends on the RAD52 inhibition in cancer cells bearing BRCA1/2 mutations or the suppression of the BRCA1-RAD51 pathway [248]. Moreover, PARP controls the epigenetic modifications of chromatin at the level of DNA and histones, and changes in PARylation modify DNA methylation patterns and directly affect DNA repair [249]. PARP inhibitors show synergistic activity to allow a selective vulnerability with DNMT1 inhibition in acute myeloid leukemia and breast cancer [250], with HDAC inhibitors in triple-negative breast cancers [251], or with the inhibition of chromatin remodeling components of the SWI/SNF (switch/sucrose non-fermentable) complex in cancer cell lines [252].

Multiple components of the DDR are also targeted in cancer stem cells that are particularly resistant to DNA damage to increase their genetic lesions and boost their chemo- and radio-sensitivities [253]. Combining PARP and ATR inhibitors, or CHK1 and WEE1 inhibitors are efficient strategies for cancer stem cells sensitization [254,255,256].

### 5.3. Targeting Replication Stress

Replication stress arises when genomic stress affects the progression of DNA polymerase and uncouples DNA polymerization from DNA unwinding. New anti-cancer therapies targeting DNA damage repair mechanisms of the DDR are developed to exploit excessive unsustainable replication stress in cancer cells having a high proliferative index compared to normal cells [226,257]. The ATR–CHK1–WEE1 signaling pathway provides the possibility to enhance fork destabilization and replicative stress, and concomitantly lowers cell cycle checkpoint thresholds and pushes tumor cells into mitotic catastrophe and cell death. A high number of approved inhibitors against CDK, or DNA repair protein such as PARP are in clinical trials one and two against advanced ovarian cancer [68]. A novel target, WRNIP1 (Werner interacting protein 1), involved in an (ATM)-dependent phosphorylation of CHK1, is implicated in counteracting an aberrant R-loop accumulation capable of blocking the replication fork [258].

The antimetabolites that incorporate into DNA in place of thymidine during the S phase, preventing nucleobase addition and terminating chain elongation, also cause DNA replication failure. Antimetabolites are pyrimidine analogs (5-fluorouracil, gemcitabine, floxuridine, or capecitabine) and purine analogs (6-mercaptopurine, 8-azaguanine, fludarabine, and cladribine) that affect DNA and RNA metabolism and arrest cells in the S phase of the cell cycle. A transcriptomic analysis of colon cancer cells’ response to 5-fluorouracil showed different cell fate phenotypes in the DNA-damage-induced genes’ expression of apoptosis and cell-cycle checkpoints [259]. Similarly, the topoisomerase poisons (forming stable cleavage complex) described in the next paragraph hamper replication and are efficient agents to increase DNA damage.

### 5.4. Replication Stress and Telomere Deprotection

Several telomere-associated factors in the shelterin complex and the CST complex (CTC1–STN1–TEN1) ensure the prevention of replication stress by capping and by regulating telomerase and DNA polymerase alpha-primase [260,261].

Telomere deprotection contributes to replication stress, induces a SAC-dependent mitotic arrest through the activation of aurora B kinase and TRF2 that dissociates from chromosomal ends, triggers structural changes from telomere-loops to linear telomeres, exposes chromosome ends to ATM, and activates the mitotic telomere DDR [65,262,263,264,265]. Deprotected telomeres induce the transcription of tDDRNAs (telomeric small non-coding DNA damage response RNAs) and subsequent DDR. The depletion of tDDRNA by complementary antisense oligonucleotides results in the inhibition of the DDR at telomeres [266]. The strategy targeting tDDRNAs with oligonucleotides could be exploited as potential biomarkers to detect the DDR activation of telomeric damage or to modulate telomeres’ function.

T-oligos (telomere homolog oligonucleotides) are homologous to the 3’ single-stranded TTAGGG overhang involved in chromosome end stabilization. They trigger telomere uncapping, or mimic DNA damage and are also promising tools. Telomere overhang oligonucleotides decrease the proliferation of prostate cancer cells [267] and non-small cell lung cancer cells [268], and sensitize mammary tumor cells to radiation [269].

Telomeric DNA G-rich sequences allow for the formation of a G-quadruplex (G4) capable of impeding the progression of the replication fork and promoting telomere fragility and replication stress [260,261,270]. Several classes of G4 interacting agents affect telomere maintenance and are promising targets for cancer therapies [271,272]. G4 ligands alter telomeric chromatin, leading to POT1 dissociation and the consequent activation of the ATR-/ATM-dependent damage pathway at the telomeres [271]. G4 ligands have been successfully developed as favorable agents (tetracarboxymethyl porphyrins) for accurate probing [273] or as specific G4 telomeric porphyrin photosensitizers (TMPipEOPP) to discriminate G-quadruplex from duplex and ssDNA [274]. Employed in combination with PARP1 inhibitor, the RHPS4 G4 ligand reduces cell proliferation, avoids uncontrolled DNA replication, and induces cell death [275]. A basal level of DNA damage and telomere deprotection increase their sensitivity in cancer cells [276]. The CX-5461 G4 ligand, a quarfloxin derivative initially identified as a RNA-Pol I inhibitor [277], was recently shown to act at transcribed regions bearing G4 structures as a DNA structure-driven TOP2 poisons [278,279]. CX-5461 induces replication-dependent DNA damage through the stabilization of G4 DNA structures, which has entered phase I/II of clinical trials for patients with BRCA1/2 deficient tumors [280] and hematologic cancers [281].

### 5.5. Other Oligonucleotides, or Small Interfering RNA

In addition to telomere homolog oligonucleotides, other oligonucleotide therapeutics based on antisense RNAs and small interfering RNA (siRNA) modify the mRNA metabolism [282]. Using siRNA against repair protein XPA (NER) and co-targeting the synthetic lethal relationship with the cell cycle checkpoints, kinase MK2 enhances the anti-tumor response in P53-deficient cancers [283]. In myeloma, ILF2 antisense oligonucleotide (Interleukin Enhancer Binding Factor, a key modulator of the DNA repair pathway) is a synthetic lethal target with DNA2 (DNA replication helicase/nuclease 2) [284]. A radiolabeled oligonucleotide that targets the RNA-associated telomerase promotes radiation-induced genomic DNA damage in telomerase-positive cancer cells [285]. A short, double-stranded oligonucleotide linked to a cholesterol molecule, AsiDNA™, acts as a decoy, mimics DSBs, and triggers a false DNA break signal to activate and attract DNA repair proteins, preventing their recruitment to the site of genomic DNA damage. The oligonucleotide acts by the overactivation of false signaling of DNA damage through DNA-PK and PARP enzymes and depletion of the DDR. The tumor DNA breaks are not repaired, accumulate damage, and drive cancer cells to death at the onset of replication [286]. The inhibition of ncRNAs overexpressing or the replacement of tumor-suppressive ncRNAs is a strategy if the inflammatory response they produce is bypassed [287].

### 5.6. Topoisomerase Inhibitors

Interference with protein-DNA complexes alters DNA structure and triggers DDR-associated cell death. Topoisomerases (Top) are involved in replication and transcription. Top1 and Top2 relax DNA superhelical tension with, respectively, SSB or DSB cleavages to allow for strand rotation before resealing the DNA. A collision between the replication fork and the cleavage complex of Top1 produces DSBs. Top covalently attaches DNA, forming reversible DNA-Top cleavage complexes during the cleavage step. From the three subgroups of human Top (type IA, IB, and IIA), types IB and IIA are particularly overexpressed in certain cancers [288]. Top inhibitors are either poisons that bind to the DNA-topoisomerase cleavage complex (interfacial inhibition) and form a ternary DNA-topoisomerase-inhibitor complex preventing the religation of the two strands of DNA; or catalytic inhibitors including DNA intercalators, ATP competitors, and inhibitors of ATP hydrolysis [289,290]. When targeted by Top poisons, the stabilization of the DNA-Top cleavage complex blocks DNA replication and generates deleterious DSBs, inducing cell death. The initial inhibitors were camptothecin (Top1 inhibitor, poison), etoposide (Top2 inhibitor, poison), and doxorubicin (Top2 inhibitor, poison) an anthracycline that intercalates, alkylates and crosslinks DNA, producing free radicals, or interferes with the helicase function. Top1 inhibitors, indenoisoquinoline derivatives LMP400 (Indotecan) and LMP776 (Indimitecan) have reached the clinical trial phase [290,291]. The only dual Top1/2 inhibitor used in therapy is aclarubicin, but other dual inhibitors exist against Top2 and other targets such as kinases or proteasome [292]. Tyrosyl-DNA phosphodiesterase 1/2 (TDP1/2) resolve Top-DNA complexes by liberating Top1 or Top2 (respectively TDP1 and TDP2) from the DNA after the degradation of the cleavage complexes. A combination of inhibitors such as the Top1 inhibitor irinotecan with a TDP1 inhibitor also improves the treatment of glioblastoma [293]. A higher response to irinotecan (Top1 inhibitor) is obtained in BRCA2-mutated cancer with acquired resistance to olaparib (PARP inhibitor) showing the downregulation of TDP1 [294]. To render the Top2 inhibitor etoposide specific for cancer cells, a covalent link was realized with a single-stranded oligonucleotide displaying a complementary sequence to a translocated cleavable DNA region only present in promyelocytic leukemia [295].

The structural optimization of Top inhibitors by glycosylation is an option to enhance the DNA damaging efficiency [296]. The addition of a carbohydrate moiety to indenoisoquinoline enhances the binding affinity of the Top1 drug to DNA due to a stronger hydrogen bonding interaction [297]. The covalent conjugation of galactose on doxorubicin increases its effect on liver cancer cells with less myocardial damage [298]. Doxorubicin uptake by the GLUT1 glucose transporters is increased by the conjugation of 2-amino-2-deoxy-D-glucose or succinic acid within cancer cells that overexpress GLUT1 and are dependent on glucose metabolism (the Warburg effect) [299].

### 5.7. DNA Damaging Drugs

The concept of increasing DNA breaks to activate the DDR protein pathway and promote cancer cell death inspired the development of various anticancer drugs. Anticancer compounds that chemically modify DNA bases, intercalate, or crosslink DNA include alkylating agents such as nitrogen mustard or platinum-derived compounds and agents that target the minor groove of DNA instead of the major groove compared to most of the alkylants [300]. Alkylating agents such as estramustine for the treatment of prostate cancer [301] or bifunctional cyclophosphamide [302] result in the covalent transfer of alkyl-groups to DNA, induce bulky DNA damage, and block transcription and replication. The anticancer activity of alkylating agents such as cyclophosphamide is in close interplay with miRNA to determine the outcome of an applied therapy [303]. Platinums such as cisplatin and its derivatives such as carboplatin, oxaliplatin, and picoplatin bind to guanine and adenine residues to form DNA adducts after the aquation of the platinum chloride group. Recently, a map of the sites of cisplatin damage and repairs for the entire human genome were obtained at a single-nucleotide resolution to better understand cancer sensitivity and resistance [304]. Other non-traditional alkylators are methylating agents, such as temozolomide used to treat glioblastoma [305]. Numerous metal-derived compounds, organometallics, are developed to generate severe DNA alterations [306,307]. Some ruthenium-containing compounds such as polynuclear ruthenium induce DNA damage and recruit DNA repair effectors such as XPC (Xeroderma Pigmentosum complementation group C) [308]. Certain organometallics target enzymes involved in DNA topology (Top1) [309]. Most metallo-glycopeptides are interesting DNA damaging drugs such as bleomycin that require a metal ion (Fe (II)) to be activated and cleave a DNA-specific sequence in 5’GT and 5’GC dinucleotides [310].

Most metal-containing compounds also belong to the class of ROS-inducing drugs that disrupt redox homeostasis, causing various types of death. Besides doxorubicin and cisplatin, a number of prooxidative drugs are used such as 2-methoxyestradiol, buthionine, or sulfoximine [311]. Cancer cells have an accelerated metabolism compared to normal cells, which generates a high level of ROS and makes them depend on MTH1 (MutT homolog 1), a repair enzyme that hydrolyzes oxidized nucleotides to corresponding monophosphates. The selective inhibition of MTH1 suppresses cancer growth through an accumulation of oxidative damage [312].

### 5.8. DNA Damaging Tools

Molecular tools are capable of introducing programmable DNA breaks. The CRISPR/Cas9 (clustered regularly interspaced palindromic repeats/associated protein 9) system provides the ability to add or remove specific DNA sequences in the genome to perform site-specific gene editing. In this plasmid-based technology, Cas9 endonuclease works guided by a single-stranded RNA at a specific site on a 20 base pair sequence among the human genome to create DSBs followed by endogenous DDR repair mechanisms NHEJ (error-prone) and HDR (high fidelity) [313]. To control DNA repair, outcomes for genome editing strategies have been developed to locally or globally inhibit or activate DNA repair effectors and favor a high-fidelity repair [314]. The fusion of DNA repair factors such as the HDR enhancer element of CtIP or Geminin to Cas9 ensures a rapid, efficient HDR [315]. Geminin inhibits the replication factor CDT1 during the S and G2 phases to avoid re-replication. The fusion of Cas9 to a dominant-negative 53BP1 inhibits NHEJ and improves HDR frequency [316]. DSB repair factors fused with Cas9 favored MMEJ and HDR in hematopoietic cells [317].

Ionizing radiation creates high levels of clustered DNA damage (including DSB, covalent crosslinking, base damage) that are difficult to repair and exploitable in radiation therapy. Over the years, radiotherapy has been improved by intensity modulation, image guided, and internal access [318,319]. The radiation efficiency is enhanced using drugs that target DNA repair machinery [320]. A new class of drugs, named radiopharmaceuticals, delivers radiation therapy specifically and directly to cancer cells. Despite the exploitation of the natural affinity of radioactive iodine to treat thyroid cancer or radium 223 to treat cancers that have spread to bones, new radiopharmaceuticals target surface molecules specifically present on cancer cells. A radiolabeled somatostatin analog compound, lutetium 177-dotatate, that targets surface receptors, is approved for the treatment of certain neuroendocrine tumors affecting the digestive tract [321], and small molecules such as PSMA (prostate-specific membrane antigen) inhibitors deliver radiotherapeutic nuclides in prostate cancer [322]. A combined strategy with an oncolytic adenovirus that inhibits the DDR, renders external beam radiation therapy more efficient [323].

## 6. Drugging DDR Proteins in Association with Other Therapies

### 6.1. Inhibition of Cell Cycle Progression and Transcription

Cancer cells are rapidly dividing cells and highly susceptible to DNA damage. Despite the fact CDKs activate the DNA damage checkpoint and initiate the DNA repair, they also interfere with the DDR. CDKs inhibition compromises CHK1 function in the DNA damage and the stalled replication [324]. The inhibition of CDK4/6 that commits the G1 and S phases’ transition inactivates the retinoblastoma tumor suppressor protein and arrests the cell cycle in G1. The CDK4/6 inhibitor, palbociclib, in combination with PARP inhibitors, added to endocrine therapy, suppresses the DNA damage repair to treat BRCA-mutated ER+ (estrogen receptor-positive) breast cancers [325]. In addition to their cell cycle regulatory functions, CDKs, especially CDK7, 8, 9, 12, and 13 phosphorylate and regulate RNA polymerase II-mediated transcription. Their inhibition can deeply affect the expression of DDR genes. CDK12 inhibitor THZ531 induces the RNA Pol II elongation defect and the premature cleavage and polyadenylation of long DDR genes followed by apoptosis in neuroblastoma cells [326]. SR-4835, a CDK12/13 inhibitor, acts in synergy with PARP inhibitors in triple-negative breast cancers [327].

### 6.2. Restoration of the P53 Pathway

P53 is the most frequently mutated gene in more than 50% of all cancers, leading to P53 protein loss or disfunction. Several strategies have been developed to reactivate P53. Murine double minute 2 (MDM2) is an E3 ubiquitin ligase responsible for P53 ubiquitination and inactivation by proteasomal degradation. MDM2 heterodimerizes with MDMX to enhance P53 ubiquitination and degradation.

Some compounds inhibit MDM2 interaction with P53, such as small molecules [328] that act as MDM2 antagonists, inhibit E3 ubiquitination of P53, or stabilize P53 and restore its conformation and DNA-binding ability [329,330]. PRIMA-1 and its analog (APR-246) restore mutated P53 proteins to a normal conformation and re-establish P53 transcriptional activity. The expression of PUMA, NOXA, and BAX are increased. PRIMA-1 is chemically converted intracellularly and binds the cysteine residues of mutated P53 protein, allowing a correct refolding [331,332]. Most P53 mutations are located in the DNA-binding core domain and create a destabilizing cavity that can be corrected by small molecules named PhiKan083 and PhiKan7088, restoring transactivation potential inducing P21 and NOXA expression with the consequent cell cycle arrest and apoptosis [333,334]. Many of those inhibitors have entered clinical trials against hematological malignancies or advanced solid tumors [335].

WIP1 phosphatase is a negative regulator of P53 after the completion of DNA repair [336]. The inhibition of WIP1 suppresses the proliferation of cancer cells by the activation of the P53 pathway. WIP1 depletion by RNA interference sensitizes cancer cells to DNA damage-inducing chemotherapy [337], and WIP1 inhibitor GSK2830371 potentiates the cytotoxic effect of doxorubicin in neuroblastoma [338].

The cell-cycle checkpoint abrogation is also a possible strategy to bypass a mutation in P53. Compared to normal cells, P53 mutated cancer cells lack a normal G1 checkpoint and mostly rely on the G2 checkpoint. The loss of WEE1 activity sensitizes P53 deficient cells to DNA damaging agents and radio sensitization [339]. The inhibition of WEE1 induces a mitotic catastrophe and cell death [340]. WEE1 inhibitor MK1775 was recently reported to suppress tumor proliferation and to potentiate Top1 inhibitor irinotecan action in P53 mutant colonic cancer cell lines [341].

### 6.3. DDR Inhibition Strategies Associated to Cancer Immunotherapies

The expression of immunosuppressive surface proteins allows cancer cells to escape immune detection and destruction. Activated T cells recognize tumor antigens, but their PD-1 receptor engaged to tumor PD-L1 (programmed death-ligand 1) or their CTLA-4 (cytotoxic T-lymphocyte associated antigen 4) interaction with B7 antigen-presenting cells inhibit T cells cytotoxicity. Consequently, antibodies against PD-1, PD-L1, or CTLA-4 are immune checkpoint blockers that potentiate anti-tumorigenic immune responses [342]. Combining a PD-L1 inhibitor with a PARP inhibitor or PARP/PD-L1 dual inhibitors [224] in breast cancers [343] allows a stronger antitumor activity compared to separate agents. In non-small cell lung cancer, pembrolizumab (anti-PD1 antibody) co-administrated with chemo- or radiotherapy showed a marked clinical benefit [344,345]. Regardless of the cancers’ tissue of origin, a large proportion of mutant neoantigens in MMR-deficient cancers are sensitive to an immune checkpoint blockade [346]. Around 200 clinical trials are testing a combination of immune checkpoint inhibitors with DNA damaging chemotherapies [226].

DNA fragments are recognized by cGAS sensor (innate immunity), followed by cGAMP synthesis, STING activation, and translocation to the Golgi, where it recruits IKKA (inhibitor of nuclear factor kappa-B kinase subunit alpha) and TBK1 (TANK-binding kinase 1) to induce the transcriptional activation of interferon and chemokines CXCL10 (chemokine (C-X-C motif) ligand 10). In addition to cytosolic DNA detection, cGAS is involved in the control of the replication dynamic by decelerating the replication fork. cGAS-deficient cells are exposed to an acceleration of the replication fork, and to replication stress that renders them hypersensitive to radiation and chemotherapy [347,348].

### 6.4. Pharmacogenomic and Predictive Markers in DDR Strategies

Cancer pharmacogenomic relates the interaction between genetic predisposition and therapeutic drugs responses [349,350]. Genome mapping of chemotherapeutic effects is performed to determine compounds selectivity for genomic regions: topotecan (Top1 inhibitor) and etoposide (Top2 inhibitor) induce similar DNA damage in transcriptional active genomic regions, daunorubicin (anthracycline) evicts histones, while aclarubicin evicts histones from heterochromatin. This different genomic specificity of DNA damage-inducing drugs has consequences for compound activity in different tumor types [351]. A prognostic signature related to the DDR pathway and a gene signature of RNA binding proteins was determined to promote an individualized treatment strategy in prostate cancer [352]. When mutated, 73 genes from the HR, FA components, and ATM/ATR kinases determine a hypersensitivity to PARP inhibitors [353]. Single nucleotide polymorphisms in ERCC1 predict the response to DNA damaging platinum chemotherapy for patients with non-small cell lung cancer [354].

The identification of markers is also important to select patients for proper therapy. A polymorphism study on CDK12, which modulates the transcription elongation of RNA polymerase II and affects the expression of DDR genes, reveals it is a possible prognostic biomarker in late-stage ovarian cancer treated with platinum [355]. Markers that predict sensitivity or resistance to induced DNA damage therapy such as the Schlaffen biomarker are useful to predict the accurate response to Top1 and Top2 inhibitors, in colon and ovarian cancers [356]. However, using a single marker to perform personalized therapy is limited and it appears necessary to integrate genomic data of several markers regarding the disease progression. The use of circulating tumor DNA (ctDNA), an emerging field in multiple cancer types, allows the evolutionary dynamics of tumors such as early stage lung cancer [357] or in bladder cancer to be tracked to follow the development of resistance in real-time using a liquid biopsy [358]. DNA-repair gene mutations are highly prevalent in ctDNA from myeloma [359]. The pharmacodynamic activation of the DDR pathway can confirm target engagement in tumors following anticancer treatment. DNA damage recognition and repair proteins (γH2AX, pS343-NBS1, and RAD51) are biomarkers detected in preclinical and clinical treatment with a quantitative multiplex immunofluorescence assay [360]. Mutation and neoantigen burden are developed as potential clinical biomarkers. The microsatellite-instability–high (MSI-H) that results from a defect in MMR in germline or somatic genes was recently approved as an indicator for immune checkpoint inhibitors [346]. Tumors with microsatellite instability respond better to PD-1 blockade with pembrolizumab in a phase two study [361]. The expression of major DDR kinases including ATM, ATR, CHK1/2, and WEE1 tailor anticancer treatment, such as systemic treatment with PD-L1-targeting monoclonal antibodies (checkpoint inhibitors). PD-L1 expression is upregulated in cancer cells in response to DSBs under genotoxic stress, such as radiotherapy or PARP inhibition. The inhibition of ATR downregulates PD-L1 in a proteasome-dependent manner, attenuates the interaction of PD-L1/PD-1, and sensitizes cancer cells to T-cell-mediated killing [362]. Using siRNA library screen targeting DNA repair genes, PD-L1 induction was shown to be dependent on the ATM/ATR/CHK1 pathway [363].

### 6.5. Viral Therapies to Boost the DDR

Viruses are actively involved in manipulating the DDR pathway targeting the MRN sensor complex of proteins for degradation and providing a rationale for their use in therapies [364]. Viral vectors have high efficiencies for gene transfer and expression. Adenoviral and retroviral vectors are primary used with tissue-specific promoters, facilitating tumor killing [365]. An adenovirus was used to express P53 (gendicine) to treat patients affected by head and neck carcinoma and is now applied to other cancers as gene therapy with adenovirus serotype-5 being the prevalent adenovirus used in clinical trials [366]. The combination of the potent Ad5/35 chimeric virus with either DNA damaging therapies (radiation or chemotherapy) leads to significant increase in the survival of aggressive tumors [367].

Oncolytic viruses such as Enadenotucirev formed as a chimera of two group B adenoviruses, Ad3 and Ad11p, are promising due to their dual action by a direct intratumor spread and oncolysis, in addition to an increase in the antitumor T-cell response [323,368]. DNA-PK inhibition sensitizes cancer cells to oncolytic alphavirus M1 and improves therapeutic effects in refractory patient tumor samples. Infection triggers the transcription of IFN and the activation of an antiviral response that is abolished by pretreatment with DNA-PK inhibitors and results in an enhanced replication of the virus within malignancies. DNA-PK inhibition further promotes viral induced DDR and increases cell apoptosis [369].

The oncolytic herpes simplex lentivirus has a unique property to target multiple components of the DDR, irrespective of their mutation. This virus generates cancer-selective sensitivity to PARP inhibitors in glioblastoma. Infection induces a loss of RAD51 due to proteasome-dependent degradation that requires viral DNA replication. This synthetic lethal-like interaction is applicable to resistant tumors in which PARP inhibitor treatments block BER and activate DDR and S phase arrest through CHK1 activation, while viral infection induces DSBs, inhibits HR repair through the degradation of RAD51, and alters the cell cycle through CHK1 degradation [370].

## 7. Conclusions

The DDR network, guardian of the genome, contains numerous proteins connected by multiple regulatory interactions. Cancers and several other diseases are caused by inherited mutations in DNA or those generated by high levels of DNA damage and repair defects in DDR proteins. To kill cancer cells, the creation of DNA-damaging agents in chemotherapy alone or combined with other agents controls the DDR to optimize anticancer activity. Choosing the right inhibitor to administer with indications from the genomic profile, and/or associated with a predictive effect of the drug would fine-tune these therapies and could adapt to the evolving profiles of tumors.
